# Selective IgA_2_ deficiency in a patient with small intestinal Crohn’s disease

**DOI:** 10.1172/JCI167742

**Published:** 2023-06-15

**Authors:** Pablo Canales-Herrerias, Yolanda Garcia-Carmona, Joan Shang, Hadar Meringer, Debra S. Yee, Lin Radigan, Sofija Buta, Gustavo Martinez-Delgado, Michael Tankelevich, Drew Helmus, Marla Dubinsky, Annelie Everts-van der Mind, Thierry Dervieux, Dusan Bogunovic, Jean-Frederic Colombel, Jason M. Brenchley, Jeremiah Faith, Charlotte Cunningham-Rundles, Andrea Cerutti, Saurabh Mehandru

**Affiliations:** 1 Precision Immunology Institute, Icahn School of Medicine at Mount Sinai, New York, New York, USA.; 2 Henry D. Janowitz Division of Gastroenterology, Department of Medicine, Icahn School of Medicine at Mount Sinai, New York, New York, USA.; 3 Barrier Immunity Section, Laboratory of Viral Diseases, National Institute of Allergy and Infectious Diseases, NIH, Bethesda, Maryland, USA.; 4 Prometheus Laboratories, San Diego, California, USA.; 5 Center for Inborn Errors of Immunity, Icahn School of Medicine at Mount Sinai, New York, New York, USA.; 6 Translational Clinical Research Program, Hospital del Mar Medical Research Institute (IMIM), Barcelona, Spain.; 7 Catalan Institute for Research and Advanced Studies (ICREA), Barcelona, Spain.

**Keywords:** Gastroenterology, Immunology, Adaptive immunity, Immunoglobulins, Inflammatory bowel disease

## To the Editor:

The human IgA response is composed of two antibody subclasses, IgA_1_ and IgA_2_. With a shorter hinge region, IgA_2_ is more resistant to bacterial proteases. Anecdotal evidence of IgA_2_ deficiency is available (1–3); however, no associations with clinical manifestations have been reported. Here, we describe a patient with selective IgA_2_ deficiency (CD068) with concomitant small intestinal Crohn’s disease (CD) and duodenal and ileal inflammation. To our knowledge, this is the first case of IgA_2_ deficiency with a potential link to inflammatory bowel disease (IBD). This report might provide insights into potential IgA_2_-specific functions.

**Figure 1:**
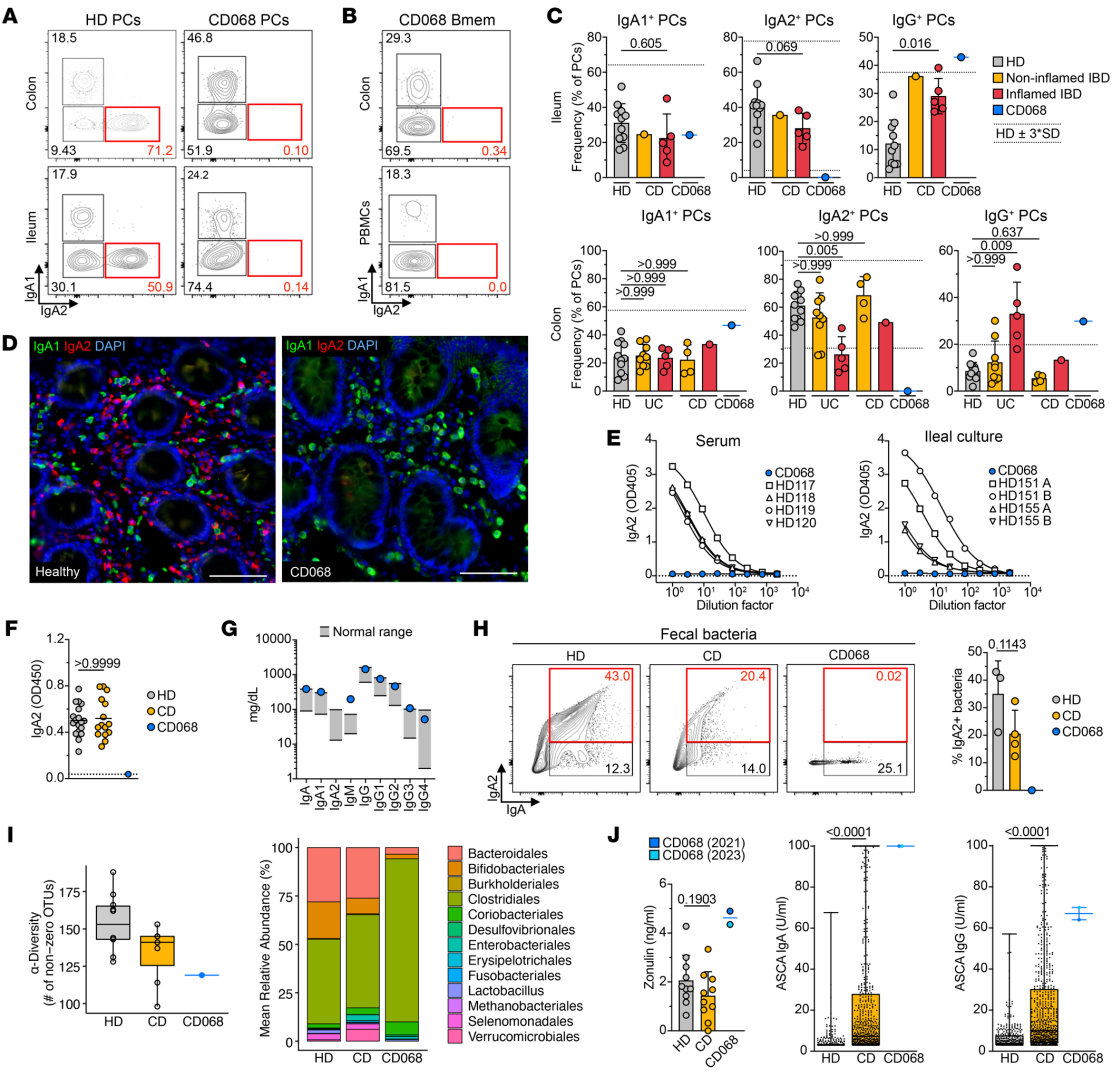
Selective IgA_2_ deficiency in a patient with small intestinal CD. Flow cytometry staining of IgA_1_ and IgA_2_ on (**A**) intestinal PCs from a representative HD and patient CD068 and (**B**) memory B (B_mem_) cells in colon and circulation from CD068. (**C**) Frequency of intestinal PCs from HDs (*n* = 19), patients with IBD (*n* = 15), and CD068. Each point represents a sample. Mean ± SD, and interval estimate are shown. (**D**) Immunofluorescence staining of IgA_1_ and IgA_2_ from a representative HD and CD068. Scale bar: 80 μm. (**E**) Secreted IgA_2_ levels in the serum and ileal tissue culture supernatant of HDs and CD068. (**F**) Plasma IgA_2_ in HDs (*n* = 18), patients with CD (*n* = 15), and CD068. (**G**) Total serum immunoglobulins from HDs (gray) and CD068. (**H**) Representative flow cytometry plots and quantification of IgA^+^ and IgA_2_^+^ microbiota in HDs (*n* = 3), patients with CD (*n* = 4), and CD068. (**I**) Metagenomic sequencing analysis from fecal samples, including α diversity and relative abundance (HD, *n* = 10; CD, *n* = 7). (**J**) Circulating zonulin and ASCA IgG and IgA in HDs (*n* = 367), patients with CD (*n* = 806), and CD068. All comparisons were done with Mann-Whitney test, except in **C**, where Kruskal-Wallis test and Dunn’s test was used. *P* values are shown.

Ileal cells, colonic cells, and PBMCs were profiled from patient CD068, 19 healthy donors (HDs), and 15 patients with IBD (Supplemental Table 1; supplemental material available online with this article; https://doi.org/10.1172/JCI167742DS1). Total absence of IgA_2_^+^ plasma cells (PCs) (Figure 1A) and switched memory B (B_mem_) cells (Figure 1B) was noted in CD068 but not in HDs. The frequency of ileal and colonic IgA_1_^+^ PCs and B_mem_ cells was comparable in CD068, patients with IBD, and HDs (Figure 1C and Supplemental Figure 1B). Conversely, a significant loss of IgA_2_^+^ PCs was detected in inflamed colonic areas from patients with UC compared with HDs, while a trend toward loss of IgA_2_^+^ cells in inflamed ileal tissues from patients with CD was noted (Figure 1C). We also found a significant expansion of IgG^+^ PCs in inflamed ilea and colons of patients with IBD compared with HDs. An increase in IgG^+^ PCs (above HD interval estimate) was observed in both inflamed (ileum) and uninflamed (colon) tissues of CD068 (Figure 1C). In a subset of individuals, paired colon/ileum samples were analyzed in parallel (Supplemental Figure 1C). Using tissue immunofluorescence, we found no IgA_2_^+^ cells in the intestinal mucosa of CD068, while IgA_1_^+^ cells were readily detected (Figure 1D). To exclude a lack of detection by our primary antibody, we stained tissue for IgA_1_ and total IgA and obtained comparable results (Supplemental Figure 1E).

In contrast to that in HDs, we could not detect IgA_2_ in the serum or ileal biopsy culture supernatants in CD068, (Figure 1E). Levels of IgA_2_ in circulation were comparable between HDs and patients with CD (Figure 1F). The concentration of all antibody isotypes except IgA_2_ was comparable between CD068 and HDs (Figure 1G). PBMCs stimulated with CD40L and IL-21 induced both IgA_1_^+^ and IgA_2_^+^ PCs in HDs, but no IgA_2_^+^ PCs were induced in CD068 (Supplemental Figure 1F). In an effort to identify a genetic basis for IgA_2_ deficiency, we used a 407-gene panel for immune deficiency, but no homozygous alterations were detected (Supplemental Table 2).

Next, we examined IgA-coated stool bacteria (Figure 1H) and found a complete lack of IgA_2_ coating. Metagenomic sequencing of stool samples in 10 HDs, 7 patients with CD, and CD068 revealed a loss of α diversity in patients with CD and CD068 compared with HDs (see Supplemental Methods). Furthermore, gut bacteria from CD068 and CD samples clustered together (Figure 1I and Supplemental Figure 1G). Finally, a distinct expansion of Clostridiales and a loss of Bacteroidales was observed in CD068. To evaluate potential consequences of intestinal dysbiosis, we measured circulating zonulin as well as IgG and IgA against *Saccharomyces cerevisiae* (ASCA) at two different time points (Figure 1J). Zonulin levels were similar in HDs and patients with CD (both *n* = 10) but were elevated in CD068. In addition, ASCA IgA and IgG concentrations from CD068 were among the highest in a large database of HDs and patients with CD (*n* = 367, *n* = 806). These data demonstrate that, in CD068, lack of IgA_2_ was associated with gut dysbiosis, impaired gut barrier integrity, and exaggerated systemic antibody responses to commensals.

Given its higher resistance to proteolysis, IgA_2_ may be particularly involved in immune exclusion, and its lack could increase epithelial penetration by commensals. Accordingly, major B cell perturbations in inflamed IBD tissue have been described previously (4), including a loss of IgA_2_^+^ PCs (5). The small intestine is the largest reservoir of IgA^+^ PCs in the body and includes bacterial communities more heavily coated by IgA than those from the colon (6), which could render the small intestine more susceptible to tissue injury due to impaired IgA (or IgA_2_) production. Although a causal relationship between the lack of IgA_2_ and CD remains unproven, we believe our study shows novel evidence that documents an association between IgA_2_ deficiency and small bowel CD with duodenal inflammation. Further studies aimed at dissecting the specific function and reactivity of gut IgA_1_ and IgA_2_ could lead to a better understanding of the contribution of IgA subclasses to IBD pathogenesis.

## Supplementary Material

Supplemental data

## Version 1 05/02/2023

In-Press Preview

## Version 2 06/15/2023

Electronic publication
