# Supplementary material for: Selective IgA2 deficiency in a patient with small intestinal Crohn’s disease
Source: J Clin Invest. 2023 Jun 15;133(12):e167742. doi: 10.1172/JCI167742 (PMC10266768; doi:10.1172/JCI167742)
Supplement: Supplemental data [file jci-133-167742-s139.pdf]

# Figure S1

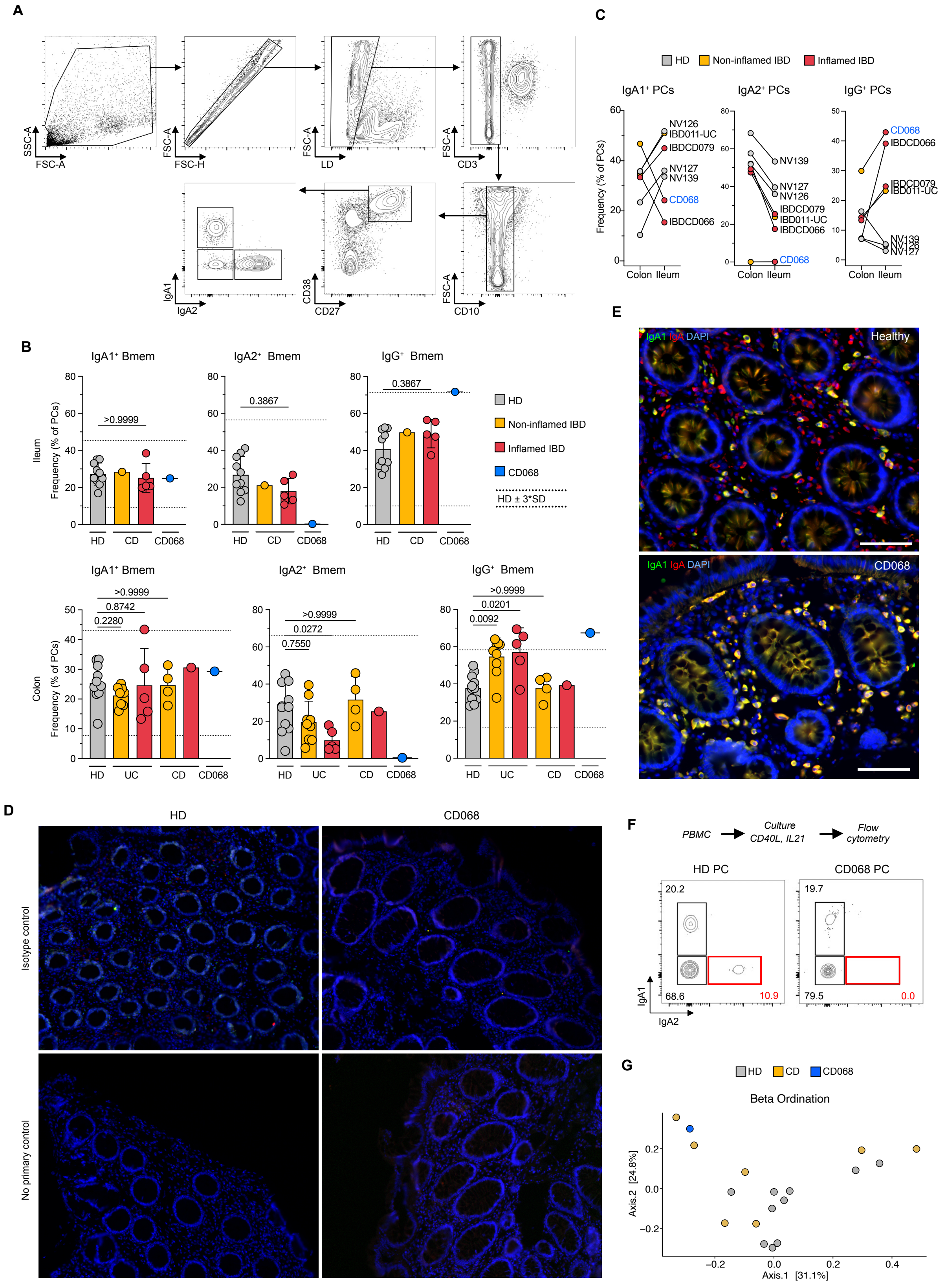

A
SSC-A
FSC-A
FSC-H
LD
CD3
CD10
CD38
CD27
IgA1
IgA2
B
IgA1+ Bmem
IgA2+ Bmem
IgG+ Bmem
Ileum
Colon
Frequency (% of PCs)
>0.9999
0.3867
0.8742
0.2280
0.0272
0.7550
0.0201
0.0092
HD
CD
UC
CD068
Non-inflamed IBD
Inflamed IBD
HD ± 3*SD
C
IgA1+ PCs
IgA2+ PCs
IgG+ PCs
Colon
Ileum
NV126
IBD011-UC
IBDCD079
NV127
NV139
IBDCD066
D
Isotype control
No primary control
E
IgA1 IgA DAPI
Healthy
F
PBMC
Culture CD40L, IL21
Flow cytometry
HD PC
CD068 PC
20.2
68.6
10.9
19.7
79.5
0.0
G
Beta Ordination
Axis.1 [31.1%]
Axis.2 [24.8%]

**Figure S1.** (A) Gating strategy used to identify viable $CD3^-CD10^-CD27^+CD38^{high}$ PCs within intestinal lamina propria mononuclear cells. (B) Frequency of intestinal $B_{mem}$ cells from HDs, noninflamed IBD patients, inflamed IBD patients, and patient CD068. (C) Frequency of intestinal PC (Figure 1C) showing paired samples taken from the same individuals. (D) Immunofluorescence (IF) analysis of isotype controls or non-primary $IgA_1$ and $IgA_2$ controls in colonic tissue sections from a representative HD (left) or patient CD068. (E) IF staining for $IgA_1$ and total IgA from a representative HD and patient CD068. (F) Representation of $IgA_1$ and $IgA_2$ on PBMC-derived PCs stimulated *in vitro* for 10 days with CD40L and IL-21. (G) Metagenomic sequencing analysis from fecal samples showing beta ordination (from Figure 1I).

# Table S1. Patient characteristics

| Study number | Group | Gender 0-female 1-male | Age (years) | Duration of Disease | Previous IBD therapies | Concomitant IBD therapy | Non-IBD medications | Disease extent (Montreal classification) | Disease behavior (Montreal classification) | Inflammation | Assay | Race | Ethnicity |
|---|---|---|---|---|---|---|---|---|---|---|---|---|---|
| IBDCD066 | CD | 0 | 41 | 1 month | none | none | ocrelizumab (MS), off for 1 year due to COVID | L3 | B1+perianal | TI | Flow cytometry | White | NA |
| IBDCD067 | CD | 0 | 20 | 2 years | none | none | none | L1+L4 | B1 | TI | Flow cytometry | White | NA |
| IBDCD068 | CD | 1 | 36 | 19 years | 5ASA, infliximab, s/p ICR, adalimumab | ustekinumab | none | L1+L4 | B2 | TI | Flow cytometry | White | not hispanic or latino |
| IBDCD070 | CD | 1 | 25 | 6 months | none | none | none | L1 | B1 | TI | Flow cytometry | White | NA |
| IBDCD072 | CD | 1 | 29 | 1.5 years | none | ustekinumab | none | L1 | B1 | TI | Flow cytometry | White | not hispanic or latino |
| IBDCD078 | CD | 1 | 54 | 32 years | steroids, s/p ICR, and repeated SB surgeries | adalimumab | lamotrigine, cariprazine, sumatriptan, atorvastatin, propranolol, fenofibrate, eszopiclone, testosterone | L3 | B2 |  | Flow cytometry | White | not hispanic or latino |
| IBDCD079 | CD | 1 | 38 | unknown | s/p ICR and colostomy > stoma reversal, | infliximab | none | L3 | B2 | TI + LC | Flow cytometry | Other | NA |
| IBDUC103 | UC | 1 | 53 | 18 years | 5ASA, steroids, infliximab, adalimumab, MTX, vedolizumab | tofacitinib | statin, clopidogrel vitamin D | E2, currently on remission |  | LC | Flow cytometry | Asian Pakist ani | NA |
| IBDUC104 | UC | 0 | 79 | 12 years | 6MP | 5ASA | amlodipine, valsartan, thiazide, statin, montelukast cetirizine | E3, currently on remission |  |  | Flow cytometry | Other | NA |
| IBDUC108 | UC | 1 | 39 | 4 years | prednisone | vedolizumab | atorvastatin, tamsulosin, PPI | E3, currently on remission |  |  | Flow cytometry | Other | NA |
| IBDUC114 | UC | 0 | 36 | 8 years | none | infliximab | none | E2, currently only mild proctitis |  |  | Flow cytometry | White | not hispanic or latino |
| IBDUC120 | UC | 1 | 38 | 1 year | steroids | 5ASA | none | E3, active moderate-severe in colon, the rest mild |  | LC | Flow cytometry | Other | NA |
| IBDUC124 | UC | 0 | 34 | 1 year | steroids 5ASA vedolizumab | infliximab | rivaroxaban | E3 pancolitis |  | LC | Flow cytometry | Asian Indian | NA |
| IBDUC128 | UC | 1 | 51 | 12 years | none | 5ASA | none | E3 pancolitis |  | LC | Flow cytometry | Asian | NA |
| UC316 | UC | 1 | 34 | 7 years | none | 5ASA | none | E2 |  | LC | Flow cytometry | Other | NA |
| IBD011 (UC) | UC | 1 | 25 | 6 years | 5ASA, infliximab, MTX | infliximab | none | left sided colitis (m/p UC and not CD) |  |  | Flow cytometry | White | NA |
| NV126 | HD | 1 | 40 | NA | NA | NA | none | NA | NA | NA | Flow cytometry | other | NA |
| NV127 | HD | 1 | 56 | NA | NA | NA | losartan, thiazide, PPI (morbid obesity, OSA, HTN) | NA | NA | NA | Flow cytometry | White | hispanic or latino (Brazil) |
| NV128 | HD | 1 | 78 | NA | NA | NA | aspirin, atorvastatin, losartan, thiazide, amlodipine (HTN, osteoarthritis, gait disorder) | NA | NA | NA | Flow cytometry | Other | NA |
| NV136 | HD | 0 | 62 | NA | NA | NA | interferon beta1, metformin, sitagliptin, statin, losartan, aspirin (HTN hyperlipidemia, multiple sclerosis, diabetes, s/p CRC and hemicolectomy) | NA | NA | NA | Flow cytometry | African Americ an | NA |
| NV137 | HD | 0 | 52 | NA | NA | NA | bupropion, levothyroxine, statin | NA | NA | NA | Flow cytometry | White | NA |
| NV138 | HD | 0 | 46 | NA | NA | NA | none (s/p breast cancer) | NA | NA | NA | Flow cytometry | African Americ an | Not hispanic or latino (Jamaican) |
| NV139 | HD | 1 | 76 | NA | NA | NA | atorvastatin, candesartan, metformin, tamsulosin, rivaroxaban, gabapentin | NA | NA | NA | Flow cytometry | Other | NA |
| NV140 | HD | 0 | 48 | NA | NA | NA | none | NA | NA | NA | Flow cytometry | White | NA |
| NV143 | HD | 1 | 50 | NA | NA | NA | amlodipine, metformin, irbesartan, statin | NA | NA | NA | Flow cytometry | Other | NA |
| NV144 | HD | 1 | 20 | NA | NA | NA | PPI (eosinophilic esophagitis) | NA | NA | NA | Flow cytometry | White | NA |
| NV146 | HD | 0 | 38 | NA | NA | NA | none | NA | NA | NA | Flow cytometry | White | NA |
| NV147 | HD | 0 | 50 | NA | NA | NA | benztropine, aripiprazole (autism) | NA | NA | NA | Flow cytometry | African Americ an | NA |
| NV148 | HD | 1 | 69 | NA | NA | NA | aspirin preventive (no concomitant disease) | NA | NA | NA | Flow cytometry | White | Not hispanic or latino |
| NV168 | HD | 1 | 59 | NA | NA | NA | PPI, atorvastatin, inhalers (dyslipidemia, GERD, OSA, COPD, prostate cancer) | NA | NA | NA | Flow cytometry | White | Not hispanic or latino |
| NV192 | HD | 1 | 43 | NA | NA | NA | none | NA | NA | NA | Flow cytometry | Other | Not hispanic or latino (domincian republic) |
| NV193 | HD | 0 | 76 | NA | NA | NA | aspirin, amlodipine, losartan, statin | NA | NA | NA | Flow cytometry | African Americ an | NA |
| NV195 | HD | 0 | 63 | NA | NA | NA | lisinopril, amlodipine, statin, PPI, thyroxine | NA | NA | NA | Flow cytometry | White | NA |
| NV196 | HD | 0 | 36 | NA | NA | NA | none | NA | NA | NA | Flow cytometry | White | NA |
| NV197 | HD | 0 | 37 | NA | NA | NA | s/p adenoid cystic carcinoma of hard palate 2018 | NA | NA | NA | Flow cytometry | Asian Indian | Not hispanic or latino |
| IBDCD002 | CD | 0 | 49 | 24 years | MTX, steroids, 5ASA, antibiotics, s/p ICR, infliximab | none (off meds d/t pancreatic cancer) | none | L3+4 | B3 |  | Plasma/Serum | White | NA |
| IBDCD007 | CD | 1 | 42 | 18 years | 5ASA, s/p surgery (ICR, segmental sigmoidectomy and takedown of vesical fistula), adalimumab, thiopurines, infliximab | ustekinumab (q4weeks) | none | L3 | B3 + perianal fistula |  | Plasma/Serum | White | NA |
| IBDCD012 | CD | 1 | 30 | 4 years | none | CDED nutrition, no medical therapy | none | L3 | inflammatory + perianal fistula |  | Plasma/Serum | White | not hispanic or latino |
| IBDCD015 | CD | 1 | 37 | 24 years | 5ASA, antibiotics, thiopurines, adalimumab, infliximab, previous surgical therapies | vedolizumab | none | L3 | B2 stricturing + perianal |  | Plasma/Serum | White | not hispanic or latino |
| IBDCD016 | CD | 0 | 50 | 6 years | 5ASA | 5ASA | none | L3 | B2 stricturing + perianal |  | Plasma/Serum | White | not hispanic or latino |
| IBDCD020 | CD | 0 | 60 | 50 years |  | thiopurines +5ASA | PPI | L2 | stricturing |  | Plasma/Serum | White | NA |
| IBDCD022 | CD | 1 | 34 | 15 years | surgery, certolizumab, infliximab, adalimumab, ustekinumab | adalimumab | none | L1+L4 | Structuring + perianal |  | Plasma/Serum | Other | NA |
| IBDCD032 | CD | 1 | 25 | 22 years | 5ASA | none | finasteride | L2 | B1 |  | Plasma/Serum | White | not hispanic or latino |
| IBDCD033 | CD | 1 | 33 | 3 years | s/p ICR and sigmoidectomy, 6MP, infliximab | ustekinumab | none | L2 | B3+perianal |  | Plasma/Serum | African Americ an | not hispanic or latino |
| IBDCD035 | CD | 0 | 67 | 24years | 5ASA, infliximab, s/p surgery (ileosigmoid fistula takedown, ileocolic resection, right colon resection, jejunocolic anastomosis, sigmoid resection) | adalimumab | levetiracetam, lorazepam, cholestyramine | L3+4 | B3 |  | Plasma/Serum | White | not hispanic or latino |
| IBDCD037 | CD | 1 | 31 | 22 years | s/p ICR, 5ASA, 6MP, infliximab | adalimumab | none | L1 | B2 |  | Plasma/Serum | White | NA |
| IBDCD040 | CD | 0 | 64 | 19 years | 5asa | none | risedronate | L2 | B3+perianal |  | Plasma/Serum | Other | Hispanic |
| IBDCD041 | CD | 1 | 45 | 6 years | infliximab, 6MP, mongersen trial | ustekinumab | losartan | L3 | B1 |  | Plasma/Serum | White | NA |
| IBDCD042 | CD | 0 | 34 | 6 years | infliximab, adalimumab, vedolizumab and tofacitinib | ustekinumab | none | L3 | B2 |  | Plasma/Serum | White | not hispanic or latino |
| IBDCD044 | CD | 0 | 38 | 18 years | 5ASA, azathioprine | infliximab | none | L2 | B1+perianal |  | Plasma/Serum | Other | NA |
| IBDCD005 | CD | 0 | 53 | 2 years | Steroids | vedolizumab | none | L1 | B2 |  | Plasma/Serum | White | NA |
| NV153 | HD | 0 | 66 | NA | NA | NA | ACE inhibitors, Aspirin, thiazide | NA | NA | NA | Plasma/Serum | African Americ an | NA |
| NV134 | HD | 1 | 59 | NA | NA | NA | aspirin, probiotics | NA | NA | NA | Plasma/Serum | White | NA |
| NV163 | HD | 0 | 45 | NA | NA | NA | Aspirin, insulin, gabapentin, nifedipine, statin | NA | NA | NA | Plasma/Serum | Other | NA |

| | | | | | | | | | | | | | |
|---|---|---|---|---|---|---|---|---|---|---|---|---|---|
| NV164 | HD | 0 | 51 | NA | NA | NA | anti histamine, PPI, eltroxin, statin | NA | NA | NA | Plasma/Serum | African American | NA |
| NV060 | HD | 0 | 48 | NA | NA | NA | none | NA | NA | NA | Plasma/Serum + Stool | African American | NA |
| NV171 | HD | 1 | 71 | NA | NA | NA | insulin , jardiance, amlodipine, statin, ACE inhibitors, carvedilol, Aspirin, fusid | NA | NA | NA | Plasma/Serum | Other | NA |
| NV162 | HD | 1 | 25 | NA | NA | NA | PPI, citalopram | NA | NA | NA | Plasma/Serum | Other | NA |
| NV172 | HD | 1 | 62 | NA | NA | NA | insulin, metformin, statin, losartan | NA | NA | NA | Plasma/Serum | Other | NA |
| NV186 | HD | 0 | 48 | NA | NA | NA | none | NA | NA | NA | Plasma/Serum | White | NA |
| NV174 | HD | 1 | 51 | NA | NA | NA | Olmesartan, amlodipine, thiazide | NA | NA | NA | Plasma/Serum | American indian or Alaskan | NA |
| NV166 | HD | 0 | 50 | NA | NA | NA | none | NA | NA | NA | Plasma/Serum | Asian | not hispanic or latino |
| NV206 | HD | 1 | 57 | NA | NA | NA | statin, levothyroxine, metformin, ezetimibe | NA | NA | NA | Plasma/Serum | White | NA |
| NV205 | HD | 0 | 33 | NA | NA | NA | spironolactone, montelukast | NA | NA | NA | Plasma/Serum | Asian | not hispanic or latino |
| NV207 | HD | 1 | 40 | NA | NA | NA | none | NA | NA | NA | Plasma/Serum | White | NA |
| NV188 | HD | 1 | 66 | NA | NA | NA | aspirin, PPI | NA | NA | NA | Plasma/Serum | Other | NA |
| NV189 | HD | 0 | 51 | NA | NA | NA | Fluoxetine, certrizine | NA | NA | NA | Plasma/Serum | Other | NA |
| NV208 | HD | 0 | 48 | NA | NA | NA | Alprazolam, certrizine, PPI, montelukast | NA | NA | NA | Plasma/Serum | Other | Latino |
| NV014 | HD | 1 | 42 | NA | NA | NA | none | NA | NA | NA | Plasma/Serum + Stool | Asian | not hispanic or latino |
| NV004 | HD | 0 | 56 | NA | NA | NA | PPI, amitryptiline, levotyroxine | NA | NA | NA | Plasma/Serum + Stool | White | NA |
| NV149 | HD | 0 | 65 | NA | NA | NA | losartan, statin, letrozole | NA | NA | NA | Plasma/Serum | Other | NA |
| NV117 | HD | 1 | 40 | NA | NA | NA | none | NA | NA | NA | Plasma/Serum | white | non-Hispanic |
| NV118 | HD | 1 | 72 | NA | NA | NA | none | NA | NA | NA | Plasma/Serum | other | Hispanic |
| NV119 | HD | 1 | 24 | NA | NA | NA | none | NA | NA | NA | Plasma/Serum | white | non-Hispanic |
| NV120 | HD | 1 | 36 | NA | NA | NA | none | NA | NA | NA | Plasma/Serum | white | Hispanic |
| NV151 | HD | 0 | 51 | NA | NA | NA | none | NA | NA | NA | Plasma/Serum | other | Hispanic |
| NV155 | HD | 0 | 60 | NA | NA | NA | none | NA | NA | NA | Plasma/Serum | other | Hispanic |
| CD1140 | CD | 0 | 21 | 14 | 5ASA, corticosteroids, Immunomodulatory drugs, metronidazole, ciprofloxacin | 5ASA 6MP | DNA | L1 | B1 + perianal | | Stool | White | not hispanic or latino |
| CD1088 | CD | 0 | 31 | 21 | 5ASA, corticosteroids, integrin blockers, canasa, SCD | vedolizumab | DNA | L3 | B1 | | Stool | White | not hispanic or latino |
| CD1039 | CD | 0 | 32 | DNA | DNA | DNA | DNA | DNA | | | Stool | White | not hispanic or latino |
| CD1084 | CD | 0 | 35 | 29 | 5ASA, corticosteroids, metronidazole, | DNA | DNA | L2 | B1 | | Stool | White | not hispanic or latino |
| CD1050 | CD | 1 | 39 | 14 | 5ASA, corticosteroids, enteral nutrition, adalimumab, ustekinumab, infliximab, bowel resection | ustekinumab | Lansoprazole | L3 | B2B3 + perianal | | Stool | White | not hispanic or latino |
| CD1107 | CD | 1 | 40 | 28 | 5ASA | DNA | loratadine/pseudoephedrine, Fluticasone, Propionate | L2 | B1 + perianal | | Stool | White | not hispanic or latino |
| CD1003 | CD | 0 | 41 | 29 | 5ASA, corticosteroids, Immunomodulatory drugs, metronidazole, ciprofloxacin | DNA | DNA | L1 | B3 | | Stool | White | not hispanic or latino |
| CD1129 | CD | 0 | 42 | DNA | DNA | DNA | DNA | DNA | | | Stool | White | not hispanic or latino |
| CD1002 | CD | 1 | 44 | 35 | 5ASA, Immunomodulatory drugs, metronidazole, ciprofloxacin | adalimumab | DNA | DNA | | | Stool | White | not hispanic or latino |
| CD1105 | CD | 0 | 52 | 21 | none | DNA | Synthroid | L3 | B2 | | Stool | White | not hispanic or latino |
| CD1081 | CD | 1 | 54 | 35 | none | Allopurinol | Colcrys | L3 | B1 | | Stool | White | not hispanic or latino |
| HD1124 | HD | 0 | 28 | NA | NA | NA | DNA | NA | NA | NA | Stool | DNA | DNA |
| HD1186 | HD | 0 | 29 | NA | NA | NA | none | NA | NA | NA | Stool | White | not hispanic or latino |
| HD1319 | HD | 1 | 29 | NA | NA | NA | none | NA | NA | NA | Stool | White | not hispanic or latino |
| HD1065 | HD | 1 | 39 | NA | NA | NA | none | NA | NA | NA | Stool | White | not hispanic or latino |
| HD1399 | HD | 1 | 43 | NA | NA | NA | none | NA | NA | NA | Stool | White | not hispanic or latino |
| 1001283B | HD | 0 | 25 | NA | NA | NA | none | NA | NA | NA | Stool | DNA | DNA |
| 1001271B | HD | 0 | 30 | NA | NA | NA | none | NA | NA | NA | Stool | DNA | DNA |
| 1001302B | HD | 0 | 49 | NA | NA | NA | none | NA | NA | NA | Stool | DNA | DNA |
| 1001275B | HD | 0 | 24 | NA | NA | NA | none | NA | NA | NA | Stool | DNA | DNA |
| 1001175B | HD | 0 | 26 | NA | NA | NA | none | NA | NA | NA | Stool | DNA | DNA |

**Table S1.** Abbreviations: 5ASA (5-aminosalicylic acid), s/p (status post), ICR (Ileocolonic resection), MTX (methotrexate), 6MP (mercaptopurine), PPI (proton pump inhibitor), OSA (obstructive sleep apnea), HTN (hypertension), CRC (colorectal cancer), GERD (gastroesophageal reflux disease), COPD (chronic obstructive pulmonary disease), CDED (Crohn's Disease Exclusion Diet), PPI (proton pump inhibitor), OSA (obstructive sleep apnea), HTN (hypertension). TI: terminal ileum, LC: left colon. NA: not applicable, DNA: data not available.

## Table S2. Genetic variants present in patient CD068

| Gene Changes | In data bases | Role | Disease associated |
|---|---|---|---|
| **TCIRG1 c.824_825del (p.Glu275Alafs*214)** | no | Pathogenic | AR osteopetrosis |
| **AP3B1 c.2389G>A (p.Val797Ile)** | no | VUS | AR Hermansky-Pudlak syndrome |
| **C7 c.2521G>C (p.Glu841Gln)** | gnomAD 0.01% | VUS | AR complement component 7 |
| **CCBE1 c.373C>T (p.Arg125Trp)** | gnomAD 0.02% | VUS | AR Hennekam lymphangiectasia-lymphedema syndrome |
| **ITGB2 c.181C>T (p.Arg61Cys)** | gnomAD 0.003%) | VUS | AR leukocyte adhesion deficiency type 1 |
| **SAMD9 c.83T>C (p.Ile28Thr)** | gnomAD 0.008%) | VUS | AD myelodysplasia, infection, restriction of growth, adrenal hypoplasia and insufficiency |
| **TCF3 c.23C>T (p.Ala8Val)** | unclear | VUS | AD/ AR and autosomal recessive agammaglobulinemia |
| **USB1 c.169G>A (p.Glu57Ly** | gnomAD 0.007% | VUS | AR poikiloderma with neutropenia |

## SUPPLEMENTAL MATERIAL

### Acknowledgements

We thank the patients who participated in the study. This work was supported by the following grants: Anonymous Donor (SM and JFC), NIH/NIDDK R01 123749 (SM and AC). We thank Michael Schwalbe for sample processing.

### Competing interests:

SM reports receiving research grants from Genentech and Takeda; receiving payment for lectures from Takeda, Genentech, Morphic; and receiving consulting fees from Takeda, Morphic, Ferring and Arena Pharmaceuticals.

JFC reports receiving research grants from AbbVie, Janssen Pharmaceuticals and Takeda; receiving payment for lectures from AbbVie, Amgen, Allergan, Inc. Ferring Pharmaceuticals, Shire, and Takeda; receiving consulting fees from AbbVie, Amgen, Arena Pharmaceuticals, Boehringer Ingelheim, BMS, Celgene Corporation, Eli Lilly, Ferring Pharmaceuticals, Galmed Research, Genentech, Glaxo Smith Kline, Janssen Pharmaceuticals, Kaleido Biosciences, Imedex, Immunic, Iterative Scopes, Merck, Microbia, Novartis, PBM Capital, Pfizer, Protagonist Therapeutics, Sanofi,Takeda, TiGenix, Vifor; and holds stock options in Intestinal Biotech Development.

## METHODS

### Patients

Patients were recruited between 2019 and 2022 in the Inflammatory Bowel Disease Center, the Gastroenterology Department and the Digestive Endoscopy Unit at Mount Sinai Hospital, NY. In accordance with the institutional review board, the protocol was approved and informed consent was obtained from all patients. Detailed patient characteristics are included in Table S1. Patients enrolled in the study were asked to donate intestinal biopsies and blood. Biopsies were collected during colonoscopies planned for regular care. When possible, ileal and colonic biopsies were obtained from the same individuals.

Patient CD068 is a 38-year-old male who had abdominal pain and bleeding at age 18, diagnosed with stricturing Crohn's disease. He had no medical history of respiratory tract infections, illnesses

or hospitalizations. His mother has a history of pemphigus vulgaris; there was no history of consanguinity. As part of our immunophenotyping efforts, intestinal biopsies were processed and characterized by flow cytometry. A 407 gene panel for immune deficiency was used to examine genetic alterations (Table S2). No homozygous alterations were noted. The patient was found to have 7 variants of unknown significance in *AP3B1, C7, CCBE1, ITGB2, SAMD9 TCF3*, and *USB1*, and one pathogenic recessive mutation in *TCIRG1* (associated with autosomal recessive osteopetrosis).

**Intestinal tissue processing**

During colonoscopy, 8-12 biopsies from non-inflamed and/or inflamed regions of the descending colon and terminal ileum were collected with forceps directly into ice-cold RPMI and transported to the laboratory. All biopsies were processed within 3h of collection. Two consecutive rounds of dissociation were performed to remove the epithelium, each consisting of 20 min incubation (37°C, 215 rpm) in 10ml HBSS (no calcium and magnesium) (Gibco) containing 5mM EDTA (Invitrogen), 100mM Hepes (Lonza) and 10% FBS, followed by 30s vortex and washing with HBSS. Biopsies were then transferred into 10ml of digestion media, made of RPMI (Gibco) containing 0.5mg/ml Collagenase IV (Sigma-Aldrich), 0.1mg/ml DNAse-1 (Roche) and 1% FBS, and were incubated for 40 min (37°C, 215rpm). Biopsies were mechanically disrupted using a syringe needle, before two filtering and washing steps (RPMI) through 100μm and 40μm cell strainers. The single cell suspension was centrifuged (400G, 10 min) and resuspended in RPMI for downstream analysis.

**Blood processing**

Blood was drawn in EDTA tubes, transported to the lab at room temperature, and processed within 3h of collection. Blood was diluted with PBS 1X (Gibco) and gently overlaid on lymphocyte separation medium (MP Biomedicals). After centrifugation, mononuclear cells were recovered and washed two times with PBS. Serum was collected and immediately stored at -80°C.

**Flow cytometry**

After isolation from tissue, cells were incubated for 20min at 4°C in PBS containing a mix of staining antibodies. Cells were then washed with PBS and fixed using PBS with 2% formaldehyde (ChemCruz) for 20 min, before washing with PBS. Cells were resuspended in PBS and acquired within 24h using LSR Fortessa (BD Bioscience). Data was analyzed using FlowJo v10 (FlowJo). Antibodies used: anti-CD19 PE Cy7 (Abcam, SJ25C1), anti-CD27 PerCP Cy5.5 (Abcam, O323),

anti-CD10 APC Cy7 (Abcam, HI10a), anti-CD38 APC (Abcam, HB-7), anti-IgD PB (Abcam, IA6-2), anti-IgM AF700 (Abcam, MHM-88), anti-CD45 PE Dazzle 594 (Abcam, HI30), anti-CD3 BV605 (Abcam, UCHT1), anti-IgA1 AF488 (Abcam, B3506B4), anti-IgA2 PE (Abcam, IS11-21E11). Interval estimate calculated as 3 X SD around the mean of control HDs.

**Tissue immunofluorescence**

Immunofluorescence staining was performed on formalin-fixed, paraffin-embedded intestinal tissue sections of 5μm thickness. Samples were deparaffinized through two 5 min xylene baths, followed by consecutive washes in solutions of decreasing concentration of ethanol in distilled water (100, 90, 80 and 70%), and two 5 min baths in PBS. Antigen retrieval was done using Dako antigen retrieval solution (Dako) and 15 min heating in pressure cooker, before washing 3x with PBS. Samples were blocked using 10% goat serum (Life technologies) or donkey serum (Southern Biotech) for 1h at room temperature. Primary antibodies were then added to the tissue in serum, and samples were incubated overnight at 4°C. Slides were washed with PBS, before adding secondary antibodies and DAPI, which were incubated for 1h at room temperature. Slides were washed with PBS and mounted using Fluoromount G (Electron Microscopy Sciences). Tissue was visualized using a Nikon Eclipse Ni microscope and a Nikon DS-Qi2 camera.
Antibodies used: rabbit anti-human IgA1 (Abcam, ab193187), mouse anti-human IgA2 (Miltenyi, 130-093-081), goat anti-human IgA (Abcam, ab98550), goat anti-mouse AF594 (Abcam, ab150116), goat anti-rabbit AF488 (Abcam, ab150077), donkey anti-goat AF594 (Abcam, ab150132), donkey anti-rabbit AF488 (Abcam, ab150073).

**Quantification of $IgA_2$ antibody levels with ELISA**

Maxisorp ELISA plates (Fischer scientific) were coated with anti-human $IgA_2$ antibody (Miltenyi) 1μg/ml in Bicarbonate buffer, and incubated for 1.5h at room temperature. After washing with PBS-Tween (PBST) (Sigma), blocking was done using PBST with 3% BSA during 1.5h at room temperature. Before being added to the plate, plasma and serum samples were diluted 1:100 in PBST-BSA, while tissue supernatant was diluted 1:3 in PBST-BSA. Serial dilutions (1:3) were made through the plate, and samples were incubated overnight at 4°C. Plates were washed 3x with PBST, and secondary anti- $IgA_2$-HRP (Southern Biotech) antibody was added and incubated 1.5h at room temperature. Finally, plates were washed with PBST and TMB substrate was added. Absorbance was measured at 450nm using a plate reader.

**Intestinal biopsy tissue culture**

During colonoscopy, biopsies were taken with forceps directly into ice-cold RPMI with 10x Antibiotic-Antimycotic (RPMI-AA) (Gibco), and transported to the lab on ice. Under sterile conditions, biopsies were rinsed on a cell strainer using RPMI-AA 10x. Biopsies were placed in pairs into 48-well plates with 1 ml RPMI-AA 10X per well, and kept from this point at 37°C with 5% $CO_2$. Culture medium was replaced every 2 days using pre-warmed RPMI-AA 2X. Recovered culture medium was centrifuged 10min at 14000G and stored at -80°C.

**Bacterial flow cytometry**

Fecal pellets were dissolved in PBS at 100 mg/ml by vortexing. Fecal slurry was centrifuged 10min at 50G to remove large particles. Supernatant was filtered through a 40nm cell strainer and cells were then washed by centrifugation at 9000G and resuspension in washing buffer (PBS, 1% BSA, 2mM EDTA). Blocking was done using PBS +1% BSA +20% mouse serum, for 20min at 4°C. Cells were stained with anti-human IgA2-PE and anti-human IgA-APC for 30min at 4°C, before washing three times with washing buffer. Samples were stained with SYBR green before analysis by flow cytometry.

**Immune globulin measurements**

For patient CD068, serum immune globulins were IgG=1,435 (nl 603 - 1,613 mg/dl); IgA=387 (nl 90-386 mg/dl); and IgM= 197 (l n20 - 172 mg/dl). IgE=13 (6-495IU/ml). In terms of IgG subclasses, IgG1- 753 (248 - 810 mg/dL) IgG2 = 464(130 - 555 mg/dL) IgG3 = 108 (15 - 102 mg/dL); IgG4 = 52 (2 - 96 mg/dl). His IgA1 = 391.6 (73.2 - 301.2 mg/dL) mg /dl, and IgA2 = <0.5 (13.4 - 97.9 mg/dL).

**Genetic Primary Immunodeficiency Panel**

A primary immune deficiency panel of 407 genes was obtained with patient's consent (Invitae Corp, San Francisco CA).

**In vitro B cell stimulation**

For B cell activation, PBMCs were cultured in RPMI media (Gibco) supplemented with 10% FBS, the presence of CD40L (1μg/ml) (InvivoGen) plus IL-21 (100 ng/ml) (BioLegend) for 10 days. Cells were kept in 24-well plates at 37°C with 5% $CO_2$. After 10 days of culture, cells were analyzed by Flow cytometry, as indicated before.

**Fecal collection, DNA extraction, and Shotgun metagenomic sequencing.**

A stool sample from the index case, CD068 was obtained and compared with stool samples from a cohort of patients with CD and HD. All samples were processed at the Microbiome Translational Center (MTC) at the Mount Sinai Hospital. First, stool samples were aliquoted on liquid nitrogen or dry ice and stored at −80°C until DNA processing. Using previously described methods, DNA was isolated from fecal samples using bead beating in phenol chloroform and prepared using NEBNext® Ultra™ II DNA Library Prep kit before samples were pooled and sequenced with Illumina HiSeq 4000 with pair-end 150nt reads (Aggarwala et al, 2021). Metagenomic samples were quality trimmed with trimmomatic (Aggarwala et al, 2021) and subsampled to a median of 1.7M reads.

**Microbiome analysis**

MetaPhlAn v2.0 (Truong et al, 2015) was utilized for taxonomic assignment of sequencing reads. Abundance table output from MetPhlAn v2.0 was used in R Studio for downstream analysis and visualizations used ggplot2 (Wickham, 2016) and ggpubr (https://github.com/kassambara/ggpubr). The *Phyloseq* package (McMurdie & Holmes, 2013) was used to integrate sample, taxonomy, and abundance taxa as well as for characterization of microbial taxonomic community with alpha diversity and principal coordinate analysis (PCoA with Bray-Curtis distances). Taxa present in less than 3% of samples, not present above 0.1% relative abundance in at least one sample or annotated as “NA” were filtered out.

**Antimicrobial antibody measurement**

Patient CD068 plasma was processed at two different time points (2021 and 2023) for specific anti-Saccharomyces cerevisiae antibodies (ASCA IgA and IgG isotypes) as described before (Plevy et al, 2013). The Prometheus Laboratories Biobank of Autoimmune (AI) and Diagnostic Immunology Data Connected to Clinical Phenotypes and Disease Characteristics was interrogated to compare the serological profile from patient CD068 with those from CD patients (n=806), and normal healthy volunteers (NHV) (n=367).

**Zonulin measurement**

Patient CD068 plasma from two time points (2021 and 2023), as well as plasma from 10 CD patients and 10 Healthy donors were used. Zonulin levels from samples were measured using the Zonulin Serum ELISA (ALPCO) kit following the manufacturer's instructions. Plasma samples were diluted 1:20 and mixed with the biotinylated zonulin family peptide (ZFP) tracer. Samples were aliquoted into the anti-ZFP coated wells in duplicate and incubated for 1 hour at room

temperature while shaking. The plate was washed five times with a plate washer and incubated with peroxidase-labelled streptavidin for 1 hour at room temperature while shaking. The plate was washed five times and incubated with tetramethylbenzidine for 15 minutes at room temperature in the dark. The stop solution was added and absorbance was measured at 450nm.

**Statistical methods**

Unpaired comparisons were done using Mann-Whitney test. When applicable, non-parametric analysis were done using Kruskal-Wallis test and Dunn's multiple comparison test. p values are shown for all comparisons. Interval estimate was calculated as 3*SD around the mean of HD values.

**Data availability**

All microbiome data generated in this study is available in BioProject (https://www.ncbi.nlm.nih.gov/bioproject/) under PRJNA637878, PRJNA946744.

**Supplementary References**


1. Aggarwala V, Mogno I, Li Z, Yang C, Britton GJ, Chen-Liaw A, Mitcham J, Bongers G, Gevers D, Clemente JC, Colombel JF, Grinspan A, Faith J. Precise quantification of bacterial strains after fecal microbiota transplantation delineates long-term engraftment and explains outcomes. Nat Microbiol. 2021 Oct;6(10):1309-1318. doi: 10.1038/s41564-021-00966-0. Epub 2021 Sep 27. Erratum in: Nat Microbiol. 2022 May;7(5):736. PMID: 34580445; PMCID: PMC8993687.
2. Truong DT, Franzosa EA, Tickle TL, Scholz M, Weingart G, Pasolli E, Tett A, Huttenhower C, Segata N. MetaPhlAn2 for enhanced metagenomic taxonomic profiling. Nat Methods. 2015 Oct;12(10):902-3. doi: 10.1038/nmeth.3589. Erratum in: Nat Methods. 2016 Jan;13(1):101. PMID: 26418763.
3. Wickham, H. (2016). Ggplot2: Elegant graphics for data analysis (2nd ed.) Springer International Publishing.
4. McMurdie PJ, Holmes S. phyloseq: an R package for reproducible interactive analysis and graphics of microbiome census data. PLoS One. 2013 Apr 22;8(4):e61217. doi: 10.1371/journal.pone.0061217. PMID: 23630581; PMCID: PMC3632530.
5. Plevy S, Silverberg MS, Lockton S, Stockfisch T, Croner L, Stachelski J, Brown M, Triggs C, Chuang E, Princen F, Singh S. Combined serological, genetic, and inflammatory markers differentiate non-IBD, Crohn's disease, and ulcerative colitis patients. Inflamm

Bowel Dis. 2013 May;19(6):1139-48. doi: 10.1097/MIB.0b013e318280b19e. PMID: 23518807; PMCID: PMC3792797.
